# Low-Dose Radiotherapy *Versus* Moderate-Dose Radiotherapy for the Treatment of Indolent Orbital Adnexal Lymphomas

**DOI:** 10.3389/fonc.2021.716002

**Published:** 2021-07-05

**Authors:** Jonathan Baron, Christopher M. Wright, Daniel Y. Lee, Maribel Carpenter, Shwetha H. Manjunath, César A. Briceño, Elise Chong, Amit Maity, John P. Plastaras, Ima Paydar

**Affiliations:** ^1^ Department of Radiation Oncology, University of Pennsylvania, Philadelphia, PA, United States; ^2^ Department of Ophthalmology, University of Pennsylvania, Philadelphia, PA, United States; ^3^ Department of Medicine, Division of Hematology-Oncology, University of Pennsylvania, Philadelphia, PA, United States

**Keywords:** orbital, indolent lymphoma, radiation, low-dose, moderate-dose, retrospective

## Abstract

**Purpose:**

Radiation therapy (RT) with doses ranging from 24 Gray (Gy) to 40 Gy is a proven treatment modality for indolent orbital adnexal lymphoma (IOAL), but recently the use of low dose RT (LDRT, defined as 2 Gy x 2 fractions) has become a notable alternative. However, limited data exists comparing outcomes following LDRT to moderate-dose RT (MDRT, RT dose 4 – 36 Gy). We present a single institution retrospective analysis comparing outcomes of patients with IOALs following LDRT or MDRT.

**Methods:**

A total of 36 patients treated with 38 consecutive courses of RT were identified; LDRT was delivered for 14 courses and MDRT for 24 courses. Overall response rates (ORR) were recorded according to Deauville or RECIST criteria with a response characterized as a complete response (CR) or partial response. Local control (LC), orbital control (OC), and overall survival (OS) rates were estimated with the Kaplan-Meier method. RT toxicity was graded per CTCAEv5 and compared with the Fisher’s exact test.

**Results:**

Median follow-up time was 29 months (m) (range, 4-129m), and median MDRT dose used was 24 Gy (range 21-36 Gy). Overall response rates (ORR) were 100% (CR 50%) and 87.5% (CR 58.3%) following LDRT and MDRT, respectively. OS at 2 years was 100% and 95% for the LDRT and MDRT groups, respectively (p=0.36). LC rates at 2 years was 100% for both LDRT and MDRT groups and at 4 years was 100% and 89% for the LDRT and MDRT groups, respectively (p=0.56). The 4-year OC rate (including both ipsilateral and contralateral relapses) was 80% and 85% for the LDRT and MDRT groups, respectively (p=0.79). No patient required treatment with RT to a previously irradiated orbit. Acute toxicities were reported following 6 LDRT courses compared to 20 MDRT courses (p=.014). No Grade 3 or higher acute toxicities occurred in either group. Late toxicities were reported following 2 LDRT courses compared to 10 MDRT courses (p=0.147).

**Conclusions:**

LDRT produced similar ORR, LC, OC, and OS rates compared to MDRT with fewer acute and minimal late toxicities reported. Future multi-center studies with larger patient numbers are warranted to show significant associations.

## Introduction

Orbital lymphomas comprise a group of uncommon and heterogenous tumors which can arise as primary disease within the orbit or as secondary disease following dissemination from an extra-orbital site (1). Although orbital lymphomas are rare, they account for the majority of orbital malignancies (2, 3). The most common type of orbital lymphomas are indolent orbital adnexal lymphomas (IOALs) with marginal zone lymphomas (MZL) and follicular lymphomas (FL) representing the most common histologies (4, 5). In general, IOALs are associated with a favorable prognosis. Prior studies have demonstrated that IOALs respond well to local radiation therapy (RT) resulting in durable local control and high survival rates (6–9).

The optimal RT dose for the treatment of IOAL has not yet been defined. Conventional doses ranging from 24 to 40 Gy resulted in excellent local control rates and were thus recommended, although some studies found that higher doses were associated with increased toxicities (6–9). Subsequently, courses of low-dose radiotherapy (LDRT) were proposed to reduce potential acute and late toxicities and were found to provide excellent short-term outcomes with minimal side-effects (10, 11). Current guidelines by the International Radiation Oncology Group (ILROG) recommend using RT doses of 24 to 25 Gy in 1.5 to 2 Gy fractions (12). Unfortunately, limited data exists directly comparing outcomes following LDRT to moderate-dose RT (MDRT) for the treatment of patients with IOALs.

Herein, we retrospectively compare outcomes including overall response rates, local control rates, orbital control rates, and overall survival for patients with IOALs treated with LDRT or MDRT.

## Methods

This study was approved by the University of Pennsylvania Institutional Review Board. A retrospective observational cohort study was performed that included all patients with IOAL treated with RT between March 2009 and October 2019 at our institution. Patients with non-indolent orbital adnexal lymphomas were excluded. Electronic medical records were reviewed for patients who met the inclusion criteria. Baseline and disease specific characteristics including sex, date of IOAL diagnosis, patient age at the beginning of RT treatment, anatomical location, unilateral or bilateral orbital involvement, maximum disease dimension, primary or secondary disease involvement, disease recurrence, date of last follow-up and survival status were recorded. Treatment information including systemic therapies used, RT start and end date, RT total dose, RT target volume, RT technique, number of RT fractions and RT toxicities were recorded when applicable. LDRT was defined as 4 Gy (2 Gy x 2 fractions) consistent with prior reports (11). MDRT was defined as 4 – 36 Gy. The use of LDRT versus MDRT was determined at the discretion of the treating physician.

Follow-up time was defined as time from start of RT to death or last follow-up. Response to treatment was graded as complete response (CR), partial response (PR), stable disease (SD) or progressive disease (PD), as defined by Deauville or RECIST criteria. Overall response rates (ORR) were characterized as achieving either a CR or PR. RT toxicity was graded by Common Terminology Criteria for Adverse Events v5.0. Acute RT toxicity was defined as ≤3 months and late toxicities as >3 months. Local recurrence was defined as recurrence within the irradiated orbit(s). Orbital recurrence was defined as recurrence within any orbital structure, either ipsilateral, contralateral, or both. Local control (LC) rates, orbital control (OC) rates, and overall survival (OS) rates were estimated with the Kaplan-Meier method from start of RT to event or last follow-up. Fisher’s exact test was used to evaluate relationships between reported toxicities following LDRT compared to MDRT. Univariate Cox regression analysis was performed for baseline characteristics associated with use of LDRT *vs* MDRT as well as for characteristics associated with the studied outcomes when applicable. A p < 0.05 was considered statistically significant. All statistical analyses were conducted using Stata 16.1 (Stata Corp, College Station, TX).

## Results

A total of 36 patients treated for IOAL were identified; individual patient characteristics are included in **Table 1**. Bilateral disease was present in 15 patients. Twenty-seven patients had primary orbital involvement, while 9 patients had secondary IOAL. The most common histology type represented was marginal zone lymphoma (MZL, 20 patients), followed by follicular lymphoma (FL, 11 patients) and mantle cell lymphoma (MCL, 5 patients).

**Table 1:** Baseline Patient Characteristics.

Characteristics	Number (Percent %)
Median Age at RT (years)	64.5, range 16-92
**Sex**	
Male	13 (36%)
Female	23 (64%)
**Disease Location**	
Unilateral	21 (58%)
Bilateral	15 (42%)
**Orbital Subsite Involved**	
Conjunctiva	7 (19%)
Lacrimal Gland	13 (36%)
Eye Lid	15 (42%)
Other	1 (3%)
**Histology**	
Marginal Zone Lymphoma	20 (56%)
Follicular Lymphoma	11 (30%)
Mantle Cell Lymphoma	5 (14%)
**Orbital Involvement**	
Primary	27 (75%)
Secondary	9 (25%)
**Systemic Therapy**	
Concurrent/Sequential Rituximab	8 (22%)
No Rituximab Use	28 (78%)
**Radiation Therapy**	
Total Courses	38 (100%)
LDRT Courses	14 (37%)
MDRT Courses	24 (63%)

The median follow-up time for all patients was 29 months (range, 4-129 months). A total of 38 radiation courses were delivered; LDRT was delivered for 14 radiation courses and MDRT for 24 radiation courses. Median MDRT dose used was 24 Gy (range 21-36 Gy) in 1.5-2 Gy fractions. Photon RT was used in 26 radiation courses (IMRT in 7 & 3D Conformal in 19), electron RT in 6 courses, and proton RT in 6 courses. Thirty courses were delivered as whole orbit RT and 8 courses as partial orbit RT. Secondary orbital involvement (odds ratio (OR) 7, p=0.021) and year of RT ≥2016 (OR 26.7, p=0.004) were associated with increased use of LDRT as compared to MDRT (**Table 2**).

**Table 2:** Univariate logistic regression for factors predicting use of LDRT *vs* MDRT and for factors predicting CR *vs* Worse Response.

	Factors Predicting LDRT *vs* MDRT		Factors Predicting CR *vs* Worse Response	
	OR (95% CI)	p-value	OR (95% CI)	p-value
**Variable**				
**Age at RT (continuous)**	1.05 (0.99-1.11)	0.093	0.99 (0.95-1.03)	0.58
**Primary Orbital Disease**				
Yes	Reference		Reference	
No	7.00 (1.34-36.69)	0.021*	1.16 (0.25-5.29)	0.847
**RT before *vs* after 2016**				
Before 2016	Reference		Reference	
2016 or After	26.71 (2.88-248.02)	0.004*	1.96 (0.52-7.41)	0.319
**Lymphoma Histology**				
Marginal Zone Lymphoma	Reference		Reference	
Follicular Lymphoma	0.33 (0.06-1.97)	0.225	1.43 (0.32-6.49)	0.642
Mantle Cell Lymphoma	1.00 (0.14-7.39)	1	0.20 (0.02-2.17)	0.188
**Bilateral Orbit Involvement**				
Yes	Reference		Reference	
No	1.69 (0.40-7.17)	0.475	0.22 (0.05-0.95)	0.042*
**LDRT *vs* MDRT**				
MDRT	NA	NA	Reference	
LDRT	NA	NA	0.51 (0.13-2.08)	0.348
**Concurrent Rituximab Therapy**				
Yes	Reference		Reference	
No	2.08 (0.16-26.96)	0.574	1.67 (0.31-8.93)	0.551

Bold values and * = p-value < .05.

**Figure 1:**
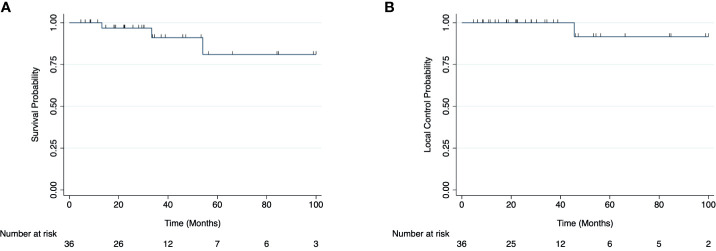
Overall survival **(A)** and local control rates **(B)** for all patients with indolent orbital adnexal lymphomas included in the cohort.

**Figure 2:**
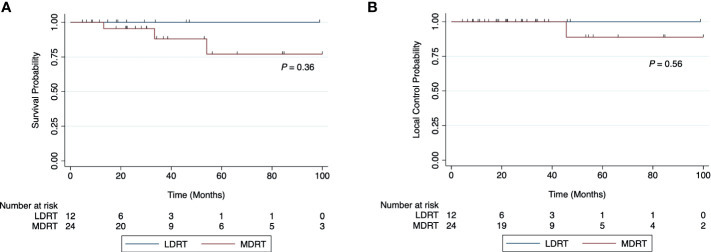
Overall survival **(A)** and local control rates **(B)** for patients with indolent orbital adnexal lymphomas treated with low-dose radiotherapy (LDRT) compared to patients treated with moderate-dose radiotherapy (MDRT).

The 2-year LC and OS rates were 100% and 95% for all patients, respectively (**Figure 1**). OS at 2 years was 100% and 95% for the LDRT and MDRT groups, respectively (**Figure 2A**, p=0.36). LC at 2 years was 100% for both LDRT and MDRT groups and at 4 years was 100% and 89% for the LDRT and MDRT groups, respectively (**Figure 2B**, p=0.56). Only 1 patient in the entire cohort experienced a local relapse. This was a patient with bilateral MZL who initially had a CR after bilateral whole orbit RT with 28 Gy in 14 fractions, but the patient experienced a relapse in the bilateral orbits 46 months after MDRT. This patient was successfully salvaged with 4 cycles of rituximab.

**Figure 3:**
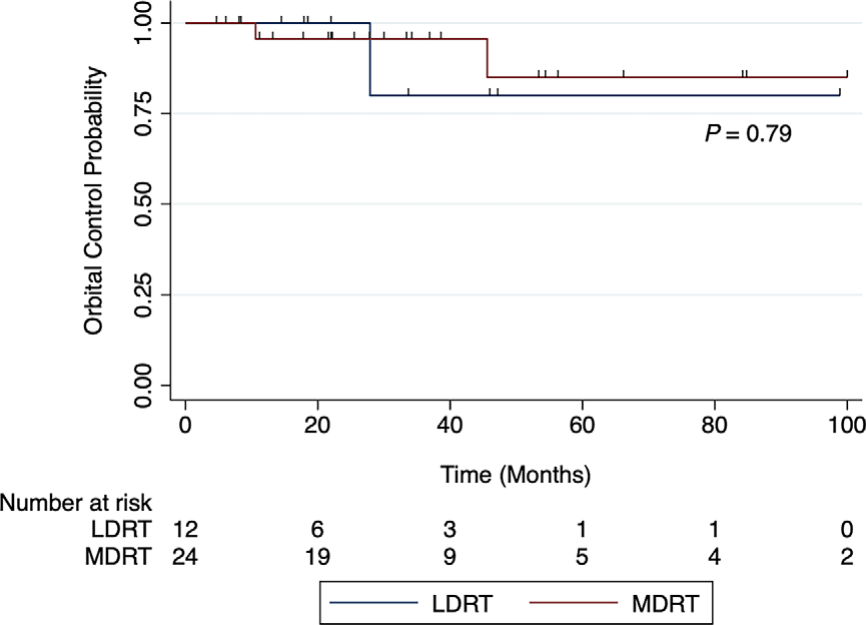
Orbital control rates for patients with indolent orbital adnexal lymphomas treated with low-dose radiotherapy (LDRT) compared to patients treated with moderate-dose radiotherapy (MDRT).

The 2-year OC rate was 100% and 96% for the LDRT and MDRT groups, respectively (**Figure 3**, p=0.79). At 4 years, the OC rate was 80% and 85% for the LDRT and MDRT groups, respectively (**Figure 3**, p=0.79). Overall, two patients in the entire cohort experienced a relapse in the contralateral orbit (1 LDRT, 1 MDRT). Both patients were retreated with LDRT and had a CR to therapy.

**Figure 4:**
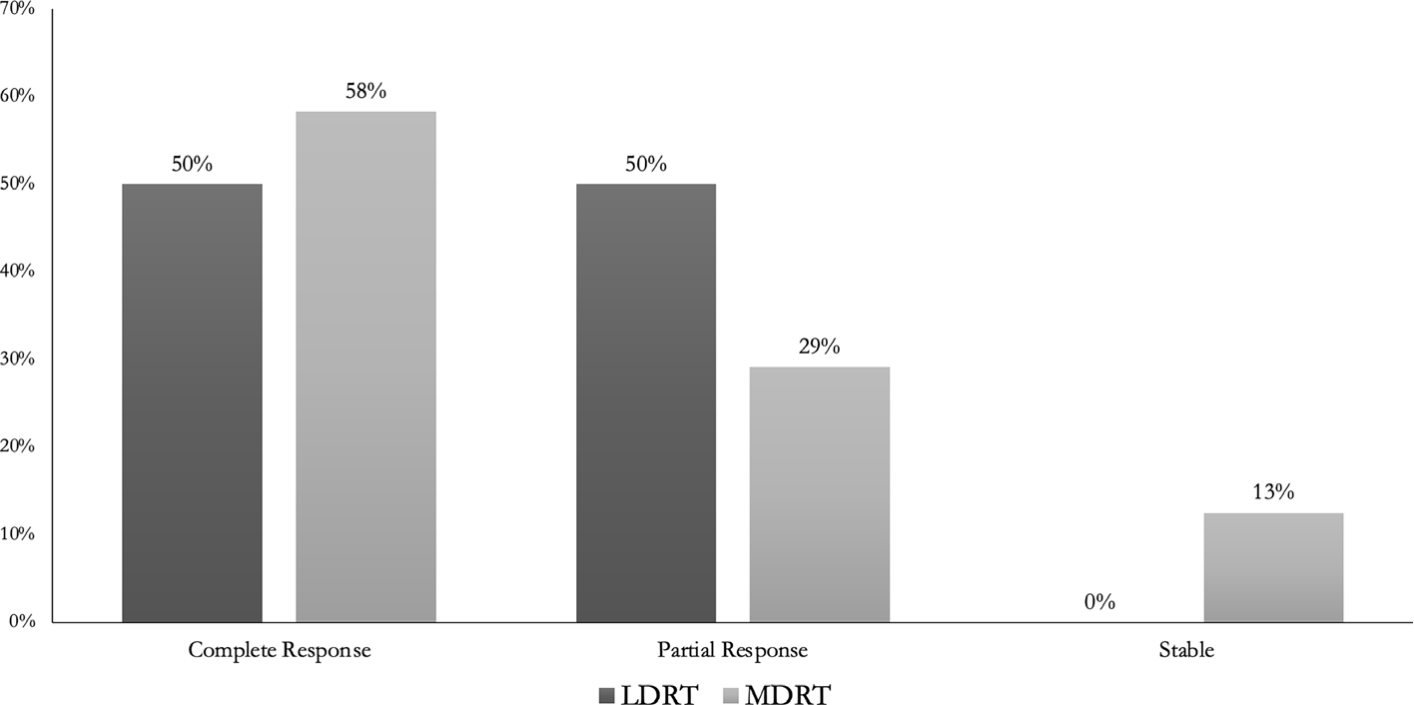
Overall Response Rates for patients with indolent orbital adnexal lymphomas treated with low-dose radiotherapy (LDRT) compared to patients treated with moderate-dose radiotherapy (MDRT).

ORR were 100% (CR 50%) and 87.5% (CR 58.3%) following LDRT and MDRT, respectively (**Figure 4**). No evaluated characteristics (for which an OR could be calculated) were significantly associated with inferior ORR (**Supplemental Table 1**). Unilateral orbital involvement was associated with inferior CR rates compared to bilateral orbital involvement (OR 0.22, p=0.042) (**Table 2**).

Acute toxicities were reported following 6 LDRT courses compared to 20 MDRT courses (p=.014). Reported individual toxicities are listed in **Table 3**. No Grade 3 (G3) or higher acute toxicities occurred in either group. Late toxicities included 8 patients with G1 dry eyes (6 MDRT, 2 LDRT), 3 patients with G2 dry eyes (3 MDRT) and one patient with a G1 cataract (1 MDRT). There were no statistically significant differences in reported late toxicities following LDRT compared to MDRT (p=0.147).

**Table 3:** Toxicities Reported Following Radiation Therapy.

Acute Toxicity	LDRT	MDRT
Fatigue	N=6 Grade 1 (6)	N=8 Grade 1 (7) Grade 2 (1)
Dermatitis	N=0	N=4 Grade 1 (3) Grade 2(1)
Headache	N=1 Grade 1 (1)	N=2 Grade 1 (2)
Erythema	N=0	N=4 Grade 1 (4)
Dry eye	N=0	N=2 Grade 1 (1) Grade 2 (1)
Other*	N=0	N=10 Grade 1 (7) Grade 2 (3)
**Late Toxicity**		
Dry eye	N=2 Grade 1 (2)	N=9 Grade 1 (6) Grade 2 (3)
Cataract	N=0	N=1 Grade 1 (1)

*Other G1 acute toxicities included reports of 2 patients with photophobia, 1 with conjunctivitis, 1 with scotomas, 1 with insomnia, 1 with eye tearing and 1 with paresthesia. Other G2 acute toxicities included 1 patient with conjunctivitis, 1 with periorbital edema, and 1 with eye tearing.

## Discussion

In this single-institution retrospective study, we describe our experience of 36 consecutive patients treated with RT for IOAL. We observed high rates of response to irradiation, durable local control, and good long term overall survival within this cohort. Furthermore, compared to more protracted RT courses, LDRT appeared to produce similar outcomes with no local recurrences or deaths. While there were no factors associated with improved ORR in the Cox regression analysis, unilateral orbital involvement was associated with inferior CR. This finding may be due to the small sample size, and a larger study will be necessary to corroborate this finding. Importantly, fewer acute toxicities were reported following LDRT compared to MDRT with no Grade 2 or higher acute toxicities reported. Also, even though we found no statistically significant differences in late toxicities following LDRT *vs* MDRT, only 2 patients (16%) treated with LDRT reported late toxicities compared to 10 patients (42%) treated with MDRT.

Conventionally, standard radiation doses of 24 – 40 Gy have been used for the treatment of this entity (6–9), but recently, the use of LDRT has become an attractive alternative. Indeed, our patients who were treated after 2016 were also more likely to receive LDRT (**Table 2**). Our data suggests that LDRT should be considered for the treatment of IOALs since the outcomes following LDRT were comparable to MDRT and fewer acute and minimal late toxicities were reported. Also, contrary to prior studies demonstrating improvements in response rates and progression free survival with higher RT doses (13), our data showed durable local control rates and response rates following LDRT. Other potential advantages of using LDRT over MDRT include lower costs of therapy and shorter duration of treatment.

Our experience comparing LDRT to MDRT is consistent with prior published data. Rehn et al., described and compared the use of LDRT, MDRT, and high-dose RT for the treatment of 45 patients with 52 ocular adnexal lymphoma lesions (14). In their study, the overall response rate across the entire cohort was 94% and no significant differences were detected in progression free survival or overall survival across the different radiation dose groups. However, more than 50% of patients included in this study were treated with high-dose RT with only 6 LDRT and 17 MDRT doses included. In comparison, our series included a greater number of both LDRT (14) and MDRT (24) doses, which are the current clinically relevant doses in the treatment of IOALs.

Our findings regarding the use of LDRT for the treatment of patients with IOALs are also similar to prior published data. Fasola et al., described the use of LDRT for the treatment of 20 patients with 27 sites of orbital adnexal NHL involvement (11). In their study, the local control rate at 2 years was 100%, and overall response rate was 96%. Similarly, Pinnix et al., described the use of LDRT in patients with indolent B‐cell and mantle cell orbital lymphomas and reported a local control rate at 2 years of 75% and an overall response rate of 100% (10). It is worth noting that although in our study the local control rate and overall response rates were comparable to the aforementioned studies, our CR rate was lower at 50%, while the other studies reported CR rates of 85% (11) and 86% (10). However, even though 50% of our patients had a PR instead of CR, none of these patients experienced a local relapse.

There were two patients in our study that had a relapse in the contralateral eye, which is a known common site of relapse for IOALs (15–17). One of these patients was initially treated with LDRT, while the other was initially treated with MDRT. Both patients were retreated with LDRT and showed a CR to therapy. Prior studies have similarly described cases of patients retreated with LDRT following a contralateral orbital relapse and achieving a CR (10, 11). This suggests a potential role for LDRT as a viable option for retreatment of patients that experience contralateral relapse regardless of the initial radiation dose. This also highlights the need to closely monitor patients with IOALs for a possible relapse in either eye.

There are several limitations to our study. First, this study was retrospective in nature so treatment assignment including radiation dose used was not randomly assigned. As a result, selection bias may be present in our study and may yield biased estimates of the treatment effects. Additionally, potentially important variables, such as the imaging technique used to determine response to therapy, reported acute and late toxicities, and the overall patient follow-up times were not standardized and may have differed among patients. Given the rarity of this condition, our single-institution study was also limited by sample size. We plan to participate in the planned multi-center collaborative review sponsored by the ILROG to further elucidate the role of LDRT in the treatment of IOALs.

## Conclusion

RT for IOALs is associated with excellent outcomes in terms of response rates and local control. Due to the exquisite radiosensitivity of IOALs, LDRT produces similar response rates and local control rates as compared to MDRT. Given the advantages of using LDRT over MDRT, our data suggests that LDRT should be considered for patients with IOAL; however, further multi-center studies with larger patient numbers are warranted.

## Data Availability Statement

The raw data supporting the conclusions of this article will be made available by the authors, without undue reservation.

## Ethics Statement

The studies involving human participants were reviewed and approved by University of Pennsylvania Institutional Review Board. Written informed consent for participation was not required for this study in accordance with the national legislation and the institutional requirements.

## Author Contributions

JB, IP, and JP conceived and designed the study. JB, CW, and MC collected and assembled the data. DL performed the statistical analysis. JB and CW wrote the manuscript. SH, CB, EC, AM, JP, and IP revised the manuscript and gave final approval. All authors contributed to the article and approved the submitted version.

## Conflict of Interest

EC reports grants from Novartis Pharmaceuticals Corporation, during the conduct of the study; personal fees from Novartis Pharmaceuticals Corporation, personal fees from Juno Therapeutics, Inc., personal fees from Kite Pharma, Inc., outside the submitted work.

The remaining authors declare that the research was conducted in the absence of any commercial or financial relationships that could be construed as a potential conflict of interest.
