# Supplementary material for: Low-Dose Radiotherapy Versus Moderate-Dose Radiotherapy for the Treatment of Indolent Orbital Adnexal Lymphomas
Source: Front Oncol. 2021 Jul 5;11:716002. doi: 10.3389/fonc.2021.716002 (PMC8288045; doi:10.3389/fonc.2021.716002)
Supplement: Supplementary file 1 [file Table_1.docx]

Supplemental Table 1: Univariate logistic regression for factors predicting overall response rate

|  | **Factors Predicting ORR** | |
| --- | --- | --- |
|  | **OR (95% CI)** | **p-value** |
| **Variable** |  |  |
| **Age at RT (continuous)** | 1.05 (.98-1.12) | 0.145 |
| **Primary Disease** |  |  |
| Yes | Colinear |  |
| No | NA | NA |
| **Year of RT** | 1.29 (.89-1.86) | 0.175 |
| **Lymphoma Histology** |  |  |
| Marginal Zone Lymphoma | Reference |  |
| Follicular Lymphoma | 1.11 (.09-13.8) | 0.935 |
| **Max Tumor Dimension** | 1.01 (.86-1.18) | 0.919 |
| **Bilateral Orbit Involvement** |  |  |
| Yes | Reference |  |
| No | 0.68 (.06-8.25) | 0.761 |
| **LDRT vs MDRT** |  |  |
| LDRT | Colinear |  |
| MDRT | NA | NA |
| **Concurrent Rituximab Therapy** |  |  |
| Yes | Reference |  |
| No | 2.17 (0.17-28.01) | 0.554 |
